# Amorphous Lycopene–PVP K30 Dispersions Prepared by Ball Milling: Improved Solubility and Antioxidant Activity

**DOI:** 10.3390/polym17212916

**Published:** 2025-10-31

**Authors:** Anna Kulawik, Maciej Kulawik, Natalia Rosiak, Wei Lu, Judyta Cielecka-Piontek, Przemysław Zalewski

**Affiliations:** 1 Poznan University of Medical Sciences, Department of Pharmacognosy and Biomaterials, Faculty of Pharmacy, Rokietnicka 3, 60-806 Poznań, Poland; 2 Poznan University of Medical Sciences, Doctoral School, Bukowska 70, 60-812 Poznań, Poland; 3 Phytopharm Klęka S.A., Klęka 1, 63-040 Nowe Miasto nad Wartą, Poland; 4 Department of Food Science and Engineering, School of Agriculture and Biology, Shanghai Jiao Tong University, Shanghai 200240, China

**Keywords:** lycopene, antioxidant, drug delivery system, bioavailability, amorphous systems

## Abstract

Lycopene is a carotenoid with strong antioxidant properties, but its therapeutic potential is limited by its poor aqueous solubility. Developing formulations that enhance its solubility and stability may improve its bioavailability and effectiveness. This study aimed to prepare amorphous lycopene–PVP K30 systems via ball milling, a solvent-free and mild technique, and to evaluate their physicochemical properties, solubility, and antioxidant activity. Formulations containing 5%, 10%, and 15% lycopene (*w*/*w*) were obtained and characterized using X-ray powder diffraction, differential scanning calorimetry, and Fourier transform infrared spectroscopy. Density Functional Theory calculations were performed to gain molecular-level insights into lycopene–polymer interactions and hydrogen-bond formation. Solubility was determined by high-performance liquid chromatography, and antioxidant activity was evaluated using the DPPH radical scavenging assay. The amorphous dispersions exhibited enhanced solubility compared to crystalline lycopene, with the 10% system showing the highest initial solubility and antioxidant capacity, while the 5% formulation demonstrated superior stability over 24 h. Ball milling proved to be an efficient method for producing amorphous lycopene–PVP K30 dispersions with improved dissolution and bioactive performance. The results indicate that lycopene loadings between 5 and 10% offer the most favorable balance between solubility, stability, and antioxidant activity, supporting their potential use in pharmaceutical formulations.

## 1. Introduction

Lycopene, a lipophilic carotenoid predominantly found in tomatoes, watermelon, pink grapefruit, and certain other fruits, has gained increasing scientific attention due to its potent antioxidant properties and broad spectrum of potential health benefits [1,2]. Its molecular structure, characterized by an extended system of conjugated double bonds, enables efficient neutralization of reactive oxygen species (ROS), including singlet oxygen, peroxyl radicals, and other free radicals, which are implicated in oxidative stress and cellular damage [3,4]. By modulating oxidative stress, lycopene contributes to the protection of critical biomolecules such as DNA, proteins, and membrane lipids [5]. Beyond its direct radical-scavenging activity, lycopene has been shown to influence key intracellular signaling pathways that are associated with antioxidant defense, inflammation, and cell survival, highlighting its potential role in the prevention and management of chronic diseases [6,7]. Epidemiological and experimental studies suggest that lycopene intake is inversely associated with the risk of cardiovascular disease, certain cancers, metabolic syndrome, and neurodegenerative conditions, emphasizing its relevance as a bioactive dietary component [3,8,9]. Additionally, lycopene exhibits anti-inflammatory, anti-proliferative, and cytoprotective effects in various in vitro and in vivo models, further extending its therapeutic potential [10].

Despite these promising biological activities, the practical application of lycopene in pharmaceutical, nutraceutical, and functional food formulations remains challenging due to its extremely low aqueous solubility and high susceptibility to environmental degradation [2,10,11,12]. Lycopene is highly lipophilic and does not dissolve in water, which significantly limits its absorption and systemic bioavailability following oral administration [13,14]. Moreover, its chemical stability is compromised under exposure to light, heat, oxygen, and acidic conditions, leading to isomerization or oxidative degradation that diminishes its biological efficacy [2,11,12]. These physicochemical limitations represent a major problem in the development of formulations that are capable of delivering lycopene consistently and effectively at therapeutically relevant concentrations. 

One promising strategy to improve the solubility of poorly water-soluble com-pounds is the generation of an amorphous form of the active substance [15,16]. Amorphization involves the disruption of the crystalline lattice, leading to the absence of long-range molecular order, which eliminates the need for additional energy to release molecules from a rigid crystal structure. As a result, the amorphous state typically exhibits superior solubility and faster dissolution rates compared with its crystalline counterpart [17,18]. However, the practical application of this approach requires the consideration of physical stability, since amorphous systems tend to revert to the more thermodynamically stable crystalline form, reducing the solubility advantage [19]. To minimize the risk of recrystallization, excipients are commonly incorporated. In particular, polymers can act as stabilizing matrices in which the active compound is dispersed, thereby enhancing the physical stability of the amorphous form [20].

Among the formulation strategies developed to overcome solubility limitations, solid dispersions (SDs) have attracted significant attention [21,22]. These systems are typically composed of one or more active pharmaceutical ingredients dispersed within an inert carrier matrix, allowing for improved wetting, porosity, and drug dispersion at the molecular level. Such modifications can enhance both solubility and the dissolution rate, thereby increasing bioavailability [23,24]. SDs can be prepared through a range of techniques, including solvent evaporation, melting, and mechanical processing. Ball milling represents a particularly straightforward and solvent-free approach, in which intimate mixing of the drug with the carrier promotes particle size reduction and may induce the partial or complete transformation of the crystalline drug into an amorphous state [25,26]. This dual effect—particle size diminution combined with amorphization—contributes to enhanced dissolution kinetics and makes ball milling a promising technique for formulating poorly water-soluble bioactives such as lycopene. In this study, the term solid dispersion refers to a molecularly dispersed amorphous system, in which lycopene molecules are homogeneously distributed within the amorphous PVP K30 matrix, rather than a simple finely divided powder.

Polymers play a key role in stabilizing amorphous systems. Polyvinylpyrrolidone (PVP), commonly referred to as povidone, is a synthetic polymer produced by radical polymerization of N-vinylpyrrolidone [27]. It is a safe, biocompatible, biodegradable, and chemically stable excipient, showing resistance to variations in temperature and pH, while at the same time demonstrating the ability to interact with both hydrophilic and lipophilic substances [27,28,29]. In physicochemical terms, PVP is an amorphous, odorless, highly hygroscopic powder, white to slightly cream in color, exhibiting colorless to almost colorless and clear solutions when dissolved in water. Its typical form is a white to off-white powder [27,30].

Depending on its molecular weight and viscosity, PVP is available in several pharmaceutical grades and is manufactured in different chemical forms [27]. The grades of PVP are identified by so-called K-values, which correspond to the Fikentscher constant and are related to the size of the polymer chains [31]. The K value determines the viscosity of the polymer solution and, indirectly, its molecular weight, constituting a key parameter in selecting the appropriate grade of PVP for a specific pharmaceutical formulation [27]. Among them, PVP K30, with an approximate molecular weight of 50 kDa, is widely applied in drug formulation [29,31]. 

Chemically, PVP is composed of repeating 1-vinyl-2-pyrrolidinone units, which give it amphiphilic properties—the ability to interact with both hydrophilic and hydrophobic molecules. Due to the presence of a polar amide group in the pyrrolidone ring and nonpolar methine and methylene groups in the polymer backbone, PVP exhibits high solubility in water, ethanol, methanol, and other organic solvents [27].

This intermediate grade combines good solubilizing properties with favorable processing characteristics, which makes it a preferred polymer for solid dispersion systems [29,31]. In aqueous environments, the viscosity and pH of PVP K30 depend on the concentration and K value, with typical 1% solutions exhibiting a pH in the range of 5.0–8.0 at 20 °C [30]. The relative viscosity of a 1% aqueous PVP K30 solution is approximately 1.241 ± 0.04 [32]. The glass transition temperature (Tg) of PVP K30 ranges from 149 to 162.5 °C, which contributes to its ability to form physically stable amorphous dispersions [33,34]. PVP K30 is characterized by excellent chemical stability in its dry form and resistance to light, oxygen, and most oxidizing agents [27].

These properties, combined with its safety of use and technological versatility, make PVP—especially its medium-molecular-weight K30 variety—one of the most frequently chosen polymers in the design of modern drug delivery systems.

PVP K30 has also been previously proposed as an efficient carrier for lycopene due to its strong solubilizing ability. Mirahmadi et al. [35] systematically screened 56 polymers and their combinations and found that polyvinylpyrrolidone K30 provided the highest solubility for lycopene among all tested materials. Their work demonstrated that PVP-based solid dispersions can effectively enhance the aqueous solubility of lycopene, providing the scientific rationale for selecting this polymer in our study.

In pharmaceutical applications, PVP plays an important role in improving the aqueous solubility, dissolution kinetics, and oral bioavailability of poorly soluble compounds [36,37]. Its capacity to form hydrogen bonds and establish stabilizing interactions with drug molecules helps maintain the amorphous form of active substances, and reduces their tendency to recrystallize [38]. Given its favorable molecular characteristics, PVP K30 is particularly attractive for formulating poorly soluble bioactives such as lycopene, where its use can significantly improve solubility and oral bioavailability.

Therefore, the aim of this study was to develop molecularly dispersed amorphous lycopene–PVP K30 systems using ball milling, in which lycopene is homogeneously distributed at the molecular level within the polymer matrix. Another aim was to evaluate the obtained system’s solubility enhancement relative to pure crystalline lycopene, as well as their antioxidant activity.

## 2. Materials and Methods

### 2.1. Materials

The study utilized lycopene (CAS No. 502-65-8, purity 98%) purchased from Angene (Nanjing, China). Polyvinylpyrrolidone K30 (PVP K30) (CAS No. 9003-39-8) was obtained from Sigma-Aldrich (St. Louis, MO, USA). High-quality laboratory water was produced using the Direct-Q 3 UV water purification system (Millipore, Molsheim, France; model Exil SA 67120). Methanol (HPLC grade) was purchased from POCH (Gliwice, Poland). 2,2-Diphenyl-1-picrylhydrazyl (DPPH) and L(+)-ascorbic acid were obtained from Sigma-Aldrich (St. Louis, MO, USA).

### 2.2. Methods

#### 2.2.1. Preparation of Lycopene Systems with PVP K30

**Figure 1:**
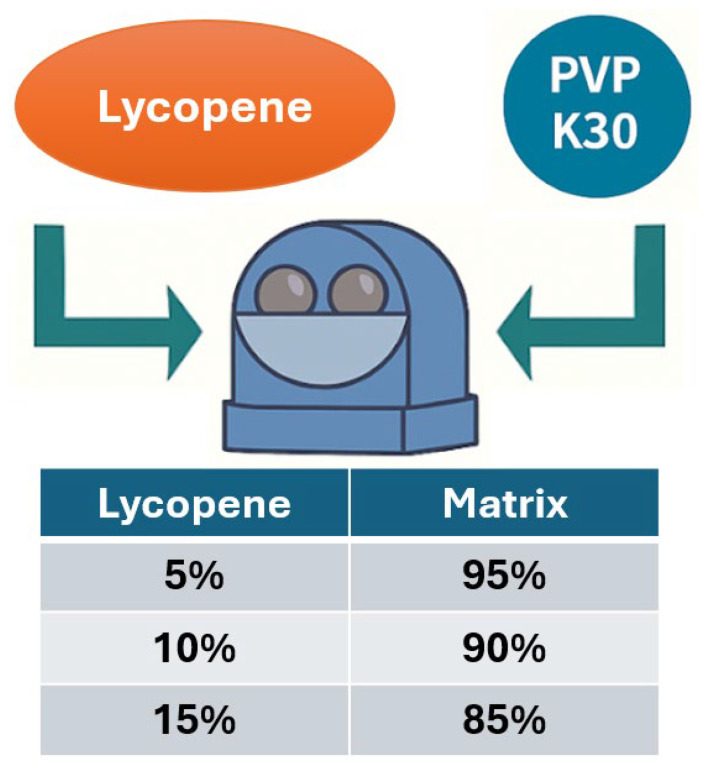
Graphical representation of the production of extrudates from lycopene and polymer matrices using the ball milling method.

Three lycopene–PVP K30 systems were prepared containing 5% (30 mg of lycopene and 570 mg of polymer), 10% (60 mg of lycopene and 540 mg of polymer), and 15% (90 mg of lycopene and 510 mg of polymer) lycopene by weight (Figure 1). For each formulation, the weighed components were placed on weighing paper using an analytical powder balance, transferred to an agate mortar, and manually ground for 10 min until a homogeneous powder was obtained. The resulting systems were then transferred to a 25 mL grinding cylinder containing two 10 mm diameter balls. Grinding was carried out in a ball mill (Mixer Mill MM 400, Retsch GmbH, Haan, Germany) at an operating frequency of 30 Hz for a total of 90 min, divided into three 30 min cycles with 10 min breaks between stages to prevent overheating. All procedures were carried out under low-light conditions. After completion, each system was transferred to an Eppendorf tube for further analysis.

#### 2.2.2. Identity Studies of Lycopene Systems with PVP K30

##### X-Ray Powder Diffraction (XRPD) Analysis

Diffraction patterns were recorded using a D2 Phaser diffractometer (Bruker AXS GmbH, Karlsruhe, Germany) equipped with a Cu Kα radiation source (λ = 1.54060 Å). The measurements were performed at an accelerating voltage of 30 kV and a current of 10 mA. The scanning range was set from 5° to 40° (2θ). For high-resolution scans, a step size of 0.02° with a counting time of 2 s per step was applied, with continuous sample rotation during data collection. A module with a fixed divergence slit of 1 mm was used for all measurements.

##### Differential Scanning Calorimetry (DSC) Analysis

Differential scanning calorimetry was employed as a complementary method to XRPD to confirm the amorphous nature of the samples. Measurements were performed using a DSC 214 Polyma calorimeter (Netzsch, Selb, Germany). Approximately 9–10 mg of each sample was weighed and sealed in aluminum pans with pierced lids. An empty aluminum pan served as the reference.

The analysis started with heating the samples from 30 °C to 150 °C at a rate of 40 °C/min. The samples were held isothermally at 150 °C for 5 min to remove moisture. Subsequently, the samples were cooled back to 30 °C at 40 °C/min. A second heating cycle was performed from 30 °C to 205 °C at a slower heating rate of 10 °C/min. After this, the samples were cooled to 5 °C at 40 °C/min, followed by a final heating cycle to 205 °C at 40 °C/min. All measurements were conducted under a nitrogen atmosphere with a constant gas flow rate of 250 mL/min.

##### Fourier Transform Infrared Spectroscopy (FTIR) Analysis

Fourier transform infrared spectroscopy was employed to evaluate chemical interactions between lycopene and the PVP K30. Spectra were recorded using an FT-IR spectrometer equipped with an attenuated total reflectance (ATR) accessory (IRTracer-100, Shimadzu, Kyoto, Japan). The spectral range was set from 4000 cm^−1^ to 400 cm^−1^ with a resolution of 4 cm^−1^, and each spectrum was the result of 400 scans.

To confirm the interactions between lycopene and the polymer, spectra of the pure substances (lycopene and PVP K30) were compared with those of the mechanical mixture and the processed system.

##### Density Functional Theory (DFT) Calculations

The lycopene 2D structure (CID: 446925) in sdf format was obtained from PubChem (https://pubchem.ncbi.nlm.nih.gov, accessed on 10 October 2025). A rough 3D geometry was generated using Avogadro 1.2.0 [39]. Gaussview 6.0.16 (Wallingford, CT, USA) was employed to draw PVP30 structures. Gaussian 16C (Wallingford, CT, USA) software was utilized to optimize the lycopene and PVP30 geometries (B3LYP/6-31 (d,p) level) and obtained theoretical FT-IR spectra of lycopene. Gaussian 16C took advantage of computational resources made available specifically via the service of “HPC cluster ‘Eagle’ for researchers,” provided through the Poznan Supercomputing and Networking Center (https://pcss.plcloud.pl, accessed on 10 October 2025).

#### 2.2.3. Evaluation of the Biological Properties of Lycopene After Creating Systems with the PVP K30

##### Solubility Study and High-Performance Liquid Chromatography (HPLC) Analysis

For each determination, 10 mg of the lycopene–PVP K30 mixtures containing 5%, 10%, and 15% lycopene (*w*/*w*) were weighed and placed in glass vials. Demineralized water (2 mL) was added. The samples were shaken at 100 rpm using a Thermo-Shaker TS-100 (BioSan, Józefów, Poland) for 2 h and 24 h at room temperature, protected from light. The solutions were then filtered through a 0.22 μm PTFE syringe filter (Merck Millipore, Darmstadt, Germany) before injection into the HPLC system.

The solubility of lycopene from the lycopene–PVP K30 systems was determined using high-performance liquid chromatography. The method has been validated. The analyses were carried out on a Shimadzu Nexera system equipped with a diode array detector (DAD) (Shimadzu Corp., Kyoto, Japan) and a 100 mm × 4.6 mm, 5 μm Dr. Maisch ReproSil-Pur Basic-C18 column (Dr. Maisch GmbH, Ammerbuch, Germany). The mobile phase consisted of 100% methanol, delivered at a flow rate of 2 mL/min. The injection volume was 10 μL, and detection was performed at 470 nm. The column temperature was maintained at 30 °C.

##### Determination of Antioxidant Activity Using the DPPH Radical Scavenging Assay

Preparation of the DPPH radical solution

The antiradical activity was assessed using a DPPH assay previously described in the literature [40]. A methanolic solution of the DPPH radical was prepared by weighing 3.9 mg of DPPH and transferring it to a 50.0 mL Erlenmeyer flask. The volume was adjusted to the mark with methanol. The flask was wrapped in aluminum foil to protect the solution from light and kept in the dark for 1 h, with occasional mixing.

Preparation of the ascorbic acid standard solution

Exactly 5.0 mg of ascorbic acid was weighed and transferred to a 10.0 mL volumetric flask wrapped in aluminum foil. The volume was made up to the mark with distilled water and thoroughly mixed. From this stock solution, 1.0 mL was diluted with distilled water to a final volume of 5.0 mL, yielding a concentration of 0.1 mg/mL.

Preparation of the standard curve

To prepare subsequent calibration standards, 1.0 mL of the more concentrated ascorbic acid solution was mixed with 1.0 mL of distilled water in 2.0 mL Eppendorf tubes wrapped in aluminum foil. The solutions were mixed thoroughly between each dilution step to obtain the following concentrations: 100.0 μg/mL, 50.0 μg/mL, 25.0 μg/mL, 12.5 μg/mL, and 6.25 μg/mL.

Preparation of test samples

Samples for antioxidant activity determination were prepared in the same manner as for the solubility study. After preparation, each sample was filtered through a 0.22 μm PTFE syringe filter. Four serial dilutions of each test solution were prepared.

DPPH radical scavenging assay

Aliquots of 25.0 μL of either the test sample or ascorbic acid standard were pipetted into the wells of a 96-well microplate. To each well, 175.0 μL of the prepared methanolic DPPH solution was added. The blank consisted of 25.0 μL of distilled water and 175.0 μL of methanol, while the control consisted of 25.0 μL of distilled water and 175.0 μL of the DPPH solution. The plates were wrapped in aluminum foil and incubated at room temperature for 30 min in a microplate thermo-shaker (TS-100, BioSan) with simultaneous mixing. Absorbance was measured at 517 nm using a microplate reader (Multiskan GO, Thermo Scientific, Waltham, MA, USA). Each measurement was performed in triplicate for all dilutions.

Antioxidant activity was calculated using the following formula:

$$DPPHscavengingactivity\left(\%\right)=\;\left[\frac{A_{o}-A_{i}}{A_{o}}\right]\times \;100\%$$

A_o_ is the absorbance of the control sample corrected by subtracting the background absorbance, and A_i_ is the absorbance of the test sample corrected in the same manner.

The half-maximal inhibitory concentration (IC_50_), defined as the antioxidant concentration required to reduce the initial DPPH radical concentration by 50%, was determined by plotting the scavenging activity (%) against the corresponding antioxidant concentration and calculating the concentration at which 50% inhibition occurred.

## 3. Results

### 3.1. Preparation of Lycopene–PVP K30 Systems

The experimental work led to the successful preparation of lycopene–PVP K30 systems using a ball milling approach. Three different formulations were obtained, containing 5%, 10%, and 15% lycopene, respectively. Obtaining such systems represents a significant achievement, since lycopene is a highly hydrophobic compound with negligible solubility in water, and its stabilization within a polymeric carrier is considered essential for improving its dissolution and functional properties. The generated formulations provided the basis for further characterization by solubility and antioxidant activity.

### 3.2. Identity Studies of Lycopene Systems with PVP K30

#### 3.2.1. XRPD Analysis

**Figure 2:**
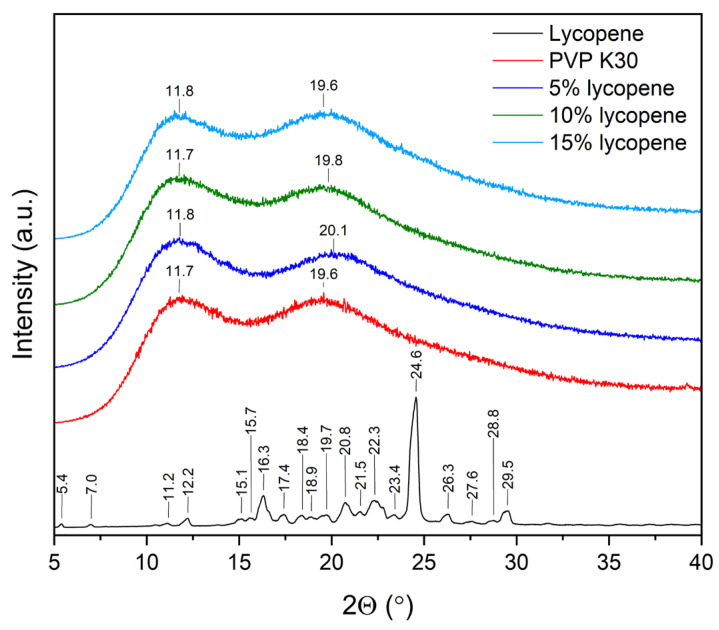
XRPD patterns of the lycopene, pure PVP K30, and tested systems.

The XRPD patterns of the lycopene, pure PVP K30, and tested systems with increasing polymer content are presented in Figure 2.

Powder X-ray diffraction was employed to confirm the disruption of the crystalline structure and the molecular dispersion of lycopene within the polymer matrix within the investigated systems. Diffractograms of pure lycopene, PVP K30, and lycopene–polymer formulations containing 5%, 10%, and 15% lycopene were recorded under identical conditions.

The diffractogram of pure lycopene displayed numerous sharp Bragg reflections within the range of 10–30° 2θ, which are indicative of its crystalline nature. In contrast, PVP K30 showed a typical amorphous pattern, characterized by a two broad halo maxima (11.8 and 19.6).

Similarly, all lycopene–PVP K30 systems (5%, 10%, and 15%) exhibited diffuse, wide scattering bands corresponding to the amorphous phase. With increasing lycopene content, a gradual increase in signal intensity was observed, yet no sharp crystalline peaks were detected. These results indicate that lycopene lost its crystalline structure upon incorporation into the polymer matrix. In addition, changes in the position of the maxima were observed. The literature indicates that the differences in the positions of the two characteristic PVP K30 maxima in amorphous solid dispersions may be related to water sorption by the sample [41,42,43].

#### 3.2.2. DSC Analysis

**Figure 3:**
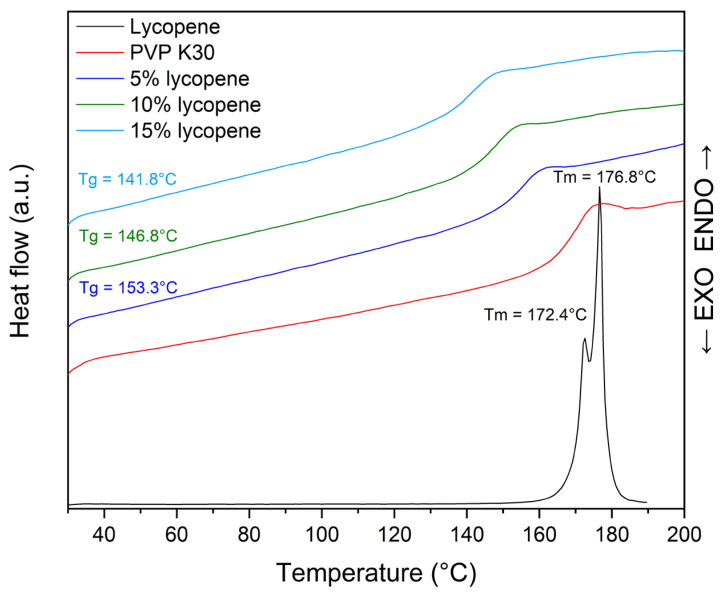
Thermogram of pure polymer, tested systems, and PVP K30.

The thermogram of the lycopene, pure PVP K30, and tested systems with increasing polymer content are presented in Figure 3.

Differential scanning calorimetry was performed as a complementary technique to XRPD to evaluate the solid-state characteristics of lycopene and its formulations with PVP K30.

The thermogram of pure lycopene (red line) displayed two distinct endothermic peaks at 172.4 °C and 176.8 °C, corresponding to its melting transitions, which are characteristic of crystalline materials. 

In the case of lycopene-polymer formulations, the disappearance of the lycopene melting peaks was accompanied by the appearance of glass transition events, confirming the amorphization of lycopene upon incorporation into the polymer matrix. The T_g_ values of the mixtures decreased with increasing lycopene content: 153.3 °C (5% lycopene, blue line), 146.8 °C (10% lycopene, green line), and 141.8 °C (15% lycopene, light blue line). This downward trend indicates a concentration-dependent modification of the thermal behavior of the system.

#### 3.2.3. FTIR Analysis and DFT Calculations

**Figure 4:**
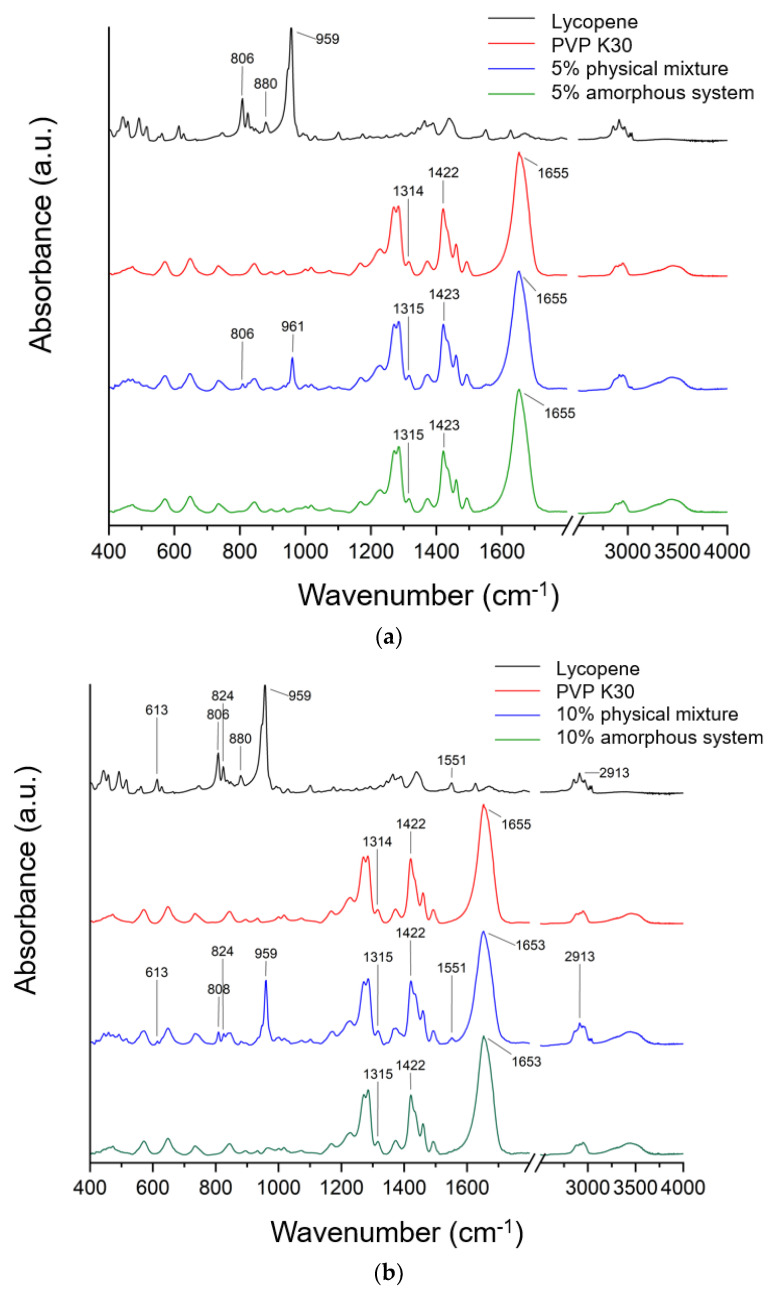

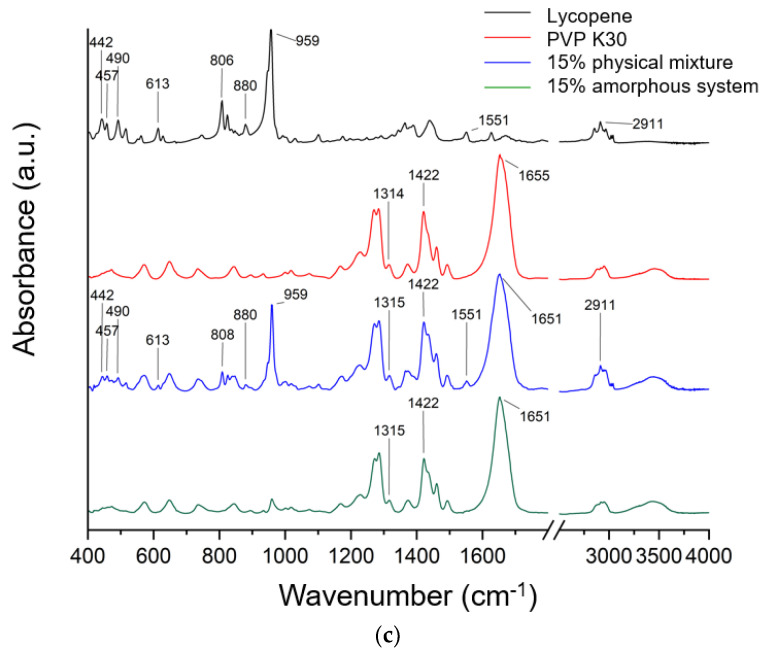
FTIR spectra recorded for lycopene, PVP K30, and (**a**) their 5% mechanical mixture and 5% amorphous system, (**b**) their 10% mechanical mixture and 10% amorphous system, and (**c**) their 15% mechanical mixture and 15% amorphous system.

FTIR spectra recorded for lycopene, PVP K30, and their mechanical mixtures and amorphous systems in tested concentrations are presented in Figure 4.

Typical lycopene FT-IR bands are CH stretching 2750–3000 cm^−1^, C-H bending in the range of 1400–1477 cm^−1^, and C-C and C-C-H stretching (1100–1400 cm^−1^). In addition, the band observed at about 959 cm^−1^ was attributed to C–H deformation vibrations, whereas the band at 613 cm^−1^ corresponded to bending vibrations of the R_2_C = CR unit within the conjugated double bond system.

The literature confirmed that in the FT-IR spectra of PVP, characteristic bands can be observed at approximately 1229 cm^−1^ (lactone structure), 1269 cm^−1^ and 1285 cm^−1^ (C–N stretching), 1373 cm^−1^ (CH_2_ bending), 1422 cm^−1^ (C–H vibrations), 1458 cm^−1^ (O–H bending), 1655 cm^−1^ (–C=O stretching of the tertiary amide in N-vinylpyrrolidone), and at 2876 cm^−1^ and 2951 cm^−1^ (C–H stretching) [41,43,44,45,46,47,48].

The spectrum of pure lycopene (black line, Figure 4) exhibited characteristic absorption bands, including a signal at 959 cm^−1^, attributed to C–H deformation vibrations, symmetric and asymmetric C–H stretching in the region of 2911–2913 cm^−1^, and a band at 613 cm^−1^ corresponding to bending vibrations of the R_2_C=CR unit within the conjugated double bond system [49,50,51].

To confirm potential interactions in amorphous systems, spectra of pure lycopene and pure PVP K30 were compared with those of the physical mixtures and the obtained systems. The spectrum of the physical mixture is a composite of bands derived from lycopene and PVP K30. The higher the lycopene content in the system, the more lycopene bands are observed in the spectrum of the physical mixture. The absence of band shifts indicates the absence of interactions in the physical mixture. In the physical mixture, the PVP K30 bands obscure most of the lycopene bands. Therefore, interactions between the individual components of the system can only be sought for the lycopene bands that are observed in a given physical mixture. For a system with 5% lycopene, these bands are observed at 806 cm^−1^ and 859 cm^−1^; for 10%, these bands are observed at 613, 808, 824, 959, 1551, and 2913 cm^−1^; while for 15%, the bands are observed at 442, 457, 490, 613, 806, 880, 959, 1551, and 2911 cm^−1^. None of these bands were observed in the obtained systems. This indicates the complete dispersion of lycopene within the polymer matrix. Similar observations were reported in our previous work, where amorphization was carried out using PVP K30 [41,43,52].

**Figure 5:**
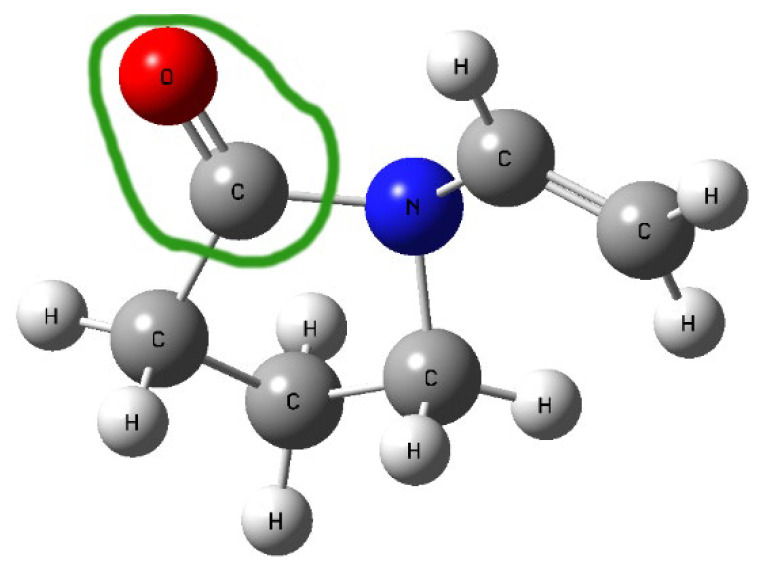
Structure of PVP K30 with the green line circling the carbonyl moiety of the pyrrolidone ring.

In the FT-IR spectra of the obtained systems, a noticeable shift in the C=O stretching band in PVP K30 (1655 cm^−1^) was observed compared to pure PVP K30. According to the literature [43,53], PVP K30 contains a single hydrogen bond acceptor group corresponding to the carbonyl moiety of the pyrrolidone ring (Figure 5).

Lycopene, as a nonpolar hydrocarbon composed only of carbon and hydrogen atoms, cannot form classical hydrogen bonds. Therefore, the observed spectral changes are most likely attributed to non-covalent interactions such as van der Waals forces, particularly of the dipole-induced dipole type, occurring between the polar C=O group of PVP K30 and the CH groups of lycopene. In addition, the disappearance or reduction of CH-related bands in the lycopene spectrum further supports the formation of intermolecular interactions between the two components, leading to system stabilization.

Density Functional Theory (DFT) analysis was employed to identify which fragments of the lycopene structure could potentially interact with PVP K30. Theoretical FT-IR bands obtained from DFT calculations were correlated with the experimental bands observed at 613, 806, 824, 880, 959, and 1551 cm^−1^ for lycopene, and the structural fragments associated with these vibrations were highlighted (Figure S1 in the Supplementary Materials). According to the theoretical spectra, the bands at 442, 457, and 490 cm^−1^ correspond to the collective vibrations of the entire molecule, while the band at 613 cm^−1^ is assigned to CCC scissoring and rocking vibrations with CH_2_ and CH_3_. The 806 cm^−1^ band arises from CH twisting, the 824 cm^−1^ band is from CH rocking and wagging vibrations, and the 880 cm^−1^ band are from CC stretching combined with CH wagging vibrations. The band at 1551 cm^−1^ is attributed to CH scissoring, wagging, and twisting vibrations.

### 3.3. Evaluation of the Biological Properties of Lycopene After Creating Systems with the PVP K30

#### 3.3.1. Lycopene Solubility—HPLC Analysis

**Figure 6:**
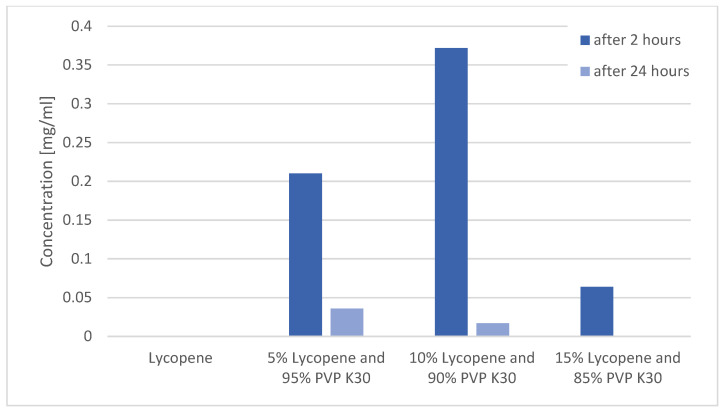
Changes in lycopene concentration in the tested systems after 2 and 24 h of shaking.

Changes in lycopene concentration in the tested systems after 2 and 24 h of shaking are presented in Figure 6.

The study confirmed that pure lycopene showed no detectable solubility in water at any of the analyzed time points (0.000 mg/mL).

After 2 h, the highest concentration of dissolved lycopene was observed for the 10% lycopene system (0.210 ± 0.010 mg/mL). A comparable value was obtained for the 5% lycopene system (0.372 ± 0.010 mg/mL). The lowest concentration was recorded for the 15% lycopene system (0.064 ± 0.008 mg/mL).

After 24 h, the solubility of all systems markedly decreased. The concentrations were 0.036 ± 0.002 mg/mL (5%), 0.017 ± 0.0015 mg/mL (10%), and 0.001 ± 0.000 mg/mL (15%).

#### 3.3.2. Antioxidant Activity—DPPH Assay

**Figure 7:**
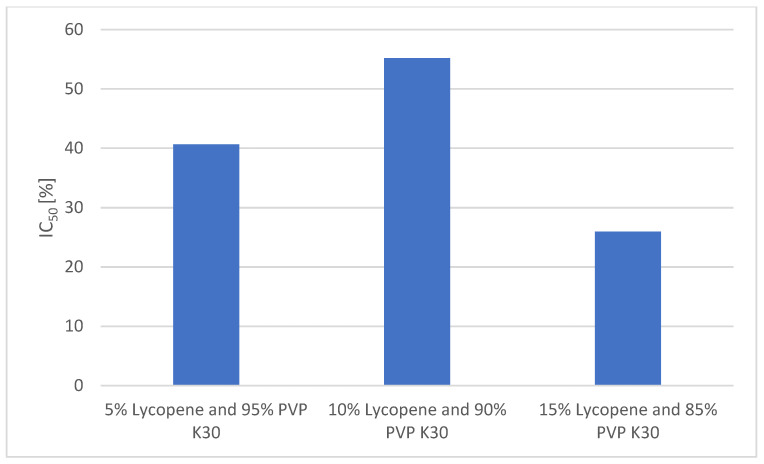
Comparison of the antioxidant activity of the formulations.

A comparison of the antioxidant activities of the formulations is presented in Figure 7.

In the DPPH radical scavenging assay, ascorbic acid (reference antioxidant) exhibited a strong dose-dependent effect, with a scavenging activity of 90.29 ± 1.23% at 1 mg/mL and 51.05 ± 1.11% at 0.5 mg/mL.

Among the lycopene–PVP K30 formulations, the highest antioxidant activity was observed for the 10% lycopene system, which achieved 55.18 ± 0.83% radical scavenging at the highest tested concentration (0.7797 mg/mL). This value corresponds to the IC_50_ of the system, indicating that over half of free radicals were neutralized at this concentration.

The 5% lycopene system showed a slightly lower activity, reaching 40.66 ± 2.96% at its highest concentration (0.210 mg/mL).

The lowest antioxidant activity was recorded for the 15% lycopene system, which achieved only 25.97 ± 0.32% scavenging at the highest tested concentration (0.1918 mg/mL).

## 4. Discussion

Our study successfully demonstrated the preparation of stable lycopene–PVP K30 molecularly dispersed amorphous systems using ball milling, resulting in significantly enhanced solubility and antioxidant activity. Although the samples were obtained as milled powders (solid–gas systems), lycopene was molecularly dispersed within the polymeric particles, forming solid solutions rather than simple physical mixtures.

According to the classification proposed by Mirahmadi et al. [35], the lycopene–PVP K30 system obtained in our study can be categorized as a type (iii) solid dispersion, in which lycopene is completely dissolved or molecularly dispersed within the polymeric matrix, as confirmed by the absence of crystalline peaks in the DSC thermogram and by FTIR evidence of molecular interactions.

The choice of PVP K30 as a polymeric excipient was of particular importance in our study. The literature evidence supports the role of this polymer in lycopene formulations prepared via other techniques. For instance, Gurav et al. [54], by using PVP K30 and Eudragit L 100, employed a solvent evaporation method to generate lyophilized polymeric nanoparticles of lycopene, while Mirahmadi et al. [35] demonstrated that mechanical mixing and solid dispersion methods using PVP K30 enhanced lycopene solubility. Similarly, Shahverdi et al. [55] prepared lycopene dispersions with PVP K30 and Poloxamer through solvent evaporation, observing significant improvements in aqueous solubility. These reports corroborate our results and highlight PVP K30 as a versatile and effective carrier for lycopene delivery.

By employing ball milling—a method not previously reported for lycopene–PVP K30 systems—our study expands the toolkit of formulation strategies for this carotenoid. The findings not only confirm the suitability of PVP K30 as a stabilizing matrix but also demonstrate that a simple, scalable, and solvent-free process can produce dispersions with improved functional properties.

XRPD analysis confirmed the successful amorphization of lycopene when processed with PVP K30. Pure lycopene exhibited a crystalline profile, as evidenced by multiple sharp reflections between 10 and 30° 2θ, in agreement with previously reported data on its crystalline form [10,56]. By contrast, the diffractograms of PVP K30 and all prepared formulations revealed broad amorphous halos, confirming the lack of long-range molecular order.

The gradual intensification of diffuse scattering with increasing lycopene concentration reflects the higher amount of lycopene incorporated into the system, yet without the reappearance of crystalline reflections. This suggests that the mechanical processing in the ball mill effectively disrupted the crystalline lattice of lycopene and promoted its molecular dispersion of lycopene in the amorphous polymer phase. The formation of such amorphous systems is advantageous, as it is often associated with improved solubility and potentially enhanced bioavailability of poorly soluble compounds like lycopene.

A similar amorphization effect was also observed for β-carotene processed with PVP and an emulsifier using hot-melt technology. XPRD analysis revealed that the crystalline signals of β-carotene almost completely disappeared, with only a broad halo characteristic of the amorphous phase remaining [57]. These findings are consistent with our results for lycopene, suggesting that polymeric carriers can effectively stabilize carotenoids that are molecularly dispersed in the polymer.

From a solid-state perspective, Ishimoto et al. [58] also confirmed through XPRD that their β-carotene dispersions prepared with polyvinylpyrrolidone (PVP, Kollidon 25) and sucrose fatty acid ester underwent complete amorphization regardless of the mixing ratio, with the crystalline reflections disappearing and only broad halos remaining. This supports the general notion that hot-melt or mechanical dispersion processes are effective in disrupting the crystal lattice of carotenoids and promoting their incorporation into an amorphous matrix.

The DSC results further corroborated the amorphous nature of lycopene in the investigated formulations, complementing the XRPD findings. Pure lycopene exhibited two melting endotherms (172.4 °C and 176.8 °C), which are consistent with its crystalline structure. Similar dual melting transitions have been previously reported for purified lycopene, with Murakami et al. [59] describing two possible melting points of purified (all-E)-lycopene at 169.48 °C and 174.38 °C, and Takehara et al. [60] reporting values of 170.66 °C and 176.35 °C. The presence of these peaks indicates a phase transition, specifically melting, which is characteristic of substances with a crystalline structure, such as lycopene. By contrast, PVP K30 displayed a glass transition at 169 °C, confirming its amorphous character.

For lycopene–PVP K30 mixtures, the disappearance of melting peaks and the emergence of glass transition signals confirmed the loss of crystalline order and the transition of lycopene into an amorphous state within the polymer matrix. The progressive reduction in T_g_ with increasing lycopene concentration suggests a plasticizing effect of lycopene or a weakening of intermolecular interactions in the polymer network.

These observations indicate that mechanical processing in the ball mill successfully disrupted the crystalline lattice of lycopene, leading to the formation of stable amorphous dispersions. The reduced T_g_ values may also imply enhanced molecular mobility within the polymeric matrix, which can be advantageous for improving the solubility and dissolution behavior of lycopene.

Comparable findings were reported by Mirahmadi et al. [35], who observed the disappearance of the lycopene melting peak in solid dispersions with PVP K30, indicating amorphization and good incorporation of lycopene into the polymer matrix. Chang et al. [61] similarly demonstrated the loss of lycopene’s melting endotherm in hot-melt dripping-pill formulations, confirming amorphization. Comparable outcomes were obtained in studies on β-carotene, where DSC analysis showed an almost complete disappearance of the melting peak corresponding to its crystalline structure, thereby confirming the formation of an amorphous dispersion [57]. This similarity underlines the crucial role of PVP in promoting and stabilizing amorphization of carotenoids. Our results therefore align with the existing literature but are distinctive in achieving complete amorphization using a solvent-free ball milling method, which provides a practical and scalable alternative. 

FTIR analysis, complementing the XRPD and DSC results, provided evidence of interactions between lycopene and the polymeric carrier. In the amorphous formulations, the disappearance of lycopene-specific bands observed in the mechanical mixtures indicates the effective disruption of the crystalline lattice and homogeneous dispersion within the polymer matrix. Additionally, changes in the polymer bands suggest that the polymer backbone has restricted vibrational freedom in the presence of lycopene, indicating potential physicochemical interactions between the two components. These observations support the successful formation of stable amorphous dispersions and highlight the role of the polymer in modulating the molecular environment of lycopene.

Overall, the FTIR findings support the hypothesis that lycopene was successfully converted into an amorphous state and molecularly dispersed within the PVP K30 matrix, with possible intermolecular interactions stabilizing the system.

Similar observations were made by Mirahmadi et al. [35], who reported band shifts and intensity changes in lycopene–PVP K30 dispersions, consistent with weak intermolecular interactions. In their study, the characteristic lycopene bands decreased in intensity but did not completely disappear, suggesting partial dispersion. In contrast, in our work the full disappearance of these bands indicates a stronger disruption of the crystalline structure and a more homogeneous molecular dispersion in the polymer matrix [35]. Chang et al. [61] also reported FTIR modifications in lycopene formulations prepared with PEG and surfactants, which they attributed to molecular interactions, further confirming the importance of polymer-based stabilization.

The results clearly demonstrate that the use of PVP K30 significantly enhanced the aqueous solubility of lycopene compared to the pure compound, which is naturally hydrophobic. Our quantitative analysis supports this claim. Pure crystalline lycopene showed no detectable solubility in water (0.000 mg/mL). In contrast, after 2 hours of incubation, lycopene dissolved from the PVP K30 systems reached 0.210 ± 0.010 mg/mL for the 5% system, 0.372 ± 0.010 mg/mL for the 10% system, and 0.064 ± 0.008 mg/mL for the 15% system. This corresponds to a substantial fold-increase relative to crystalline lycopene, clearly demonstrating that incorporation into PVP K30 significantly enhances aqueous solubility.

At the 2-hour time point, the 10% lycopene system exhibited the highest solubility. However, this system also showed the greatest decline in lycopene concentration after 24 hours, suggesting limited stability over time. The 5% lycopene system, although showing a slightly lower initial solubility, retained a higher proportion of lycopene after 24 hours, indicating potentially greater stability of the complex.

The lowest solubility at both time points was observed for the 15% lycopene system, which may indicate that the saturation limit of the polymer–lycopene complex had been exceeded, leading to precipitation. It should be emphasized that this decrease in solubility at the highest lycopene loading is not due to an intrinsic solubility-lowering effect of PVP K30. Rather, it reflects the exceeding of the polymer’s stabilization capacity, resulting in partial precipitation, aggregation, or dissociation of the complex over time. This highlights the importance of carefully optimizing the polymer-to-lycopene ratio: while PVP effectively enhances the initial solubility of hydrophobic carotenoids, excessive lycopene content relative to the polymer can compromise both solubility and stability.

The observed decline in lycopene concentration over time may be attributed to precipitation, complex dissociation, or particle aggregation in the aqueous medium. From the perspective of potential applications in dietary supplements or pharmaceutical formulations, the 5% and 10% systems appear to be more promising in terms of initial solubility, although further optimization is required to improve their stability.

Mirahmadi et al. [35] also found that PVP K30 improves lycopene solubility but noted that dissolution efficiency varied with carrier type and lycopene loading. Excessive lycopene concentrations sometimes led to precipitation and lower overall dissolution, which is consistent with our observation that 15% lycopene dispersions are less stable. Similar trends were also observed by Prosapio et al. [62], who investigated the coprecipitation of β-carotene with PVP using a supercritical antisolvent process. Their findings confirmed that the polymer substantially accelerated the dissolution of the carotenoid, with microparticle formulations prepared using lower-molecular-weight PVP showing up to a tenfold faster dissolution compared to unprocessed β-carotene. Importantly, they noted that the polymer-to-carotenoid ratio was critical: excessive amounts of PVP led to particle coalescence and suboptimal morphology, while too high a carotenoid load reduced stability and recovery. These results further support the notion that an optimal balance between lycopene concentration and carrier content is essential to maximize the solubility and stability of the formulations. Taken together, both studies highlight that while PVP is a highly effective solubilizer for hydrophobic carotenoids, careful optimization of the drug-to-polymer ratio is required to avoid precipitation or instability over time.

It is worth noting that in the case of β-carotene prepared by the hot-melt method with PVP and an emulsifier, an even greater increase in aqueous solubility was achieved, reaching approximately 120 µg/mL [57]. These results indicate that the combined presence of a polymer and surface-active agents not only enhances the amorphization process but also improves carotenoid dispersion in aqueous media and prevents recrystallization. Although emulsifiers were not employed in our lycopene systems, the observed solubility improvement with PVP K30 fits into this trend. Further optimization of the formulation could therefore enhance both the solubility and stability of lycopene in water.

In another study, Ishimoto et al. [58] demonstrated that the dissolution ratio of β-carotene in amorphous solid dispersions strongly depends on the drug-to-carrier ratio. Their formulations, prepared with polyvinylpyrrolidone (PVP, Kollidon 25) and sucrose fatty acid ester as carriers, showed that lowering the proportion of carotenoid relative to the excipients markedly increased the dissolution fraction, with dispersions containing a less active compound achieving dissolution ratios above 50%, while higher drug loadings led to much poorer solubility outcomes. Importantly, they established a clear correlation between the amount of compound dissolved in water and its oral bioavailability in rats, underscoring that maximizing the fraction in solution is critical to ensuring absorption [58]. These findings directly parallel our results with lycopene, where higher concentrations beyond 10% appeared to exceed the stabilization capacity of the polymer, leading to precipitation and reduced solubility.

The DPPH assay results confirm that the incorporation of lycopene into PVP K30 matrices enhances its radical scavenging activity compared to the naturally low aqueous solubility of free lycopene. The 10% lycopene formulation displayed the highest activity, reaching an IC_50_ at 0.7797 mg/mL. This suggests that an optimal polymer-to-lycopene ratio facilitates better dispersion and accessibility of lycopene molecules to react with free radicals.

Interestingly, while the 5% lycopene formulation demonstrated lower maximum activity than the 10% system, its performance was still substantial, indicating that effective antioxidant functionality can be retained at reduced lycopene loading. It should be noted that the measured antioxidant activity in the DPPH assay is influenced not only by the intrinsic antioxidant potential of lycopene but also by its solubility and molecular accessibility in the reaction medium. Despite its strong intrinsic radical scavenging properties, the 5% lycopene formulation contains fewer molecules that are available to interact with DPPH radicals compared with higher-load systems, and its lipophilic nature may limit interaction with the predominantly aqueous DPPH solution. Therefore, although the 5% system retains substantial activity, it appears to be lower than that of ascorbic acid, which is fully soluble and highly reactive in the assay medium. This highlights that the observed differences in radical scavenging reflect both formulation-dependent dispersion and assay-specific limitations rather than a reduction in the intrinsic antioxidant potential of lycopene.

The markedly lower activity of the 15% lycopene formulation may be explained by the reduced dispersion efficiency and potential aggregation of lycopene at high loading, which could limit the surface area available for radical interaction.

Overall, the results suggest that moderate lycopene loadings (5–10%) in PVP K30 are more favorable for maximizing antioxidant performance than high loadings (15%). These findings are consistent with the solubility data, indicating that both antioxidant activity and stability may benefit from avoiding the supersaturation of the polymer carrier.

It should be noted that while the current study demonstrates the enhanced solubility and antioxidant activity of lycopene in PVP K30 systems, it has certain limitations. No in vitro digestion, bioavailability, cytotoxicity, or safety assessments were performed. Therefore, the results primarily reflect the physicochemical and radical-scavenging properties of the formulations. Future studies should aim to evaluate the performance of these systems under simulated gastrointestinal conditions, assess oral bioavailability, and investigate cytotoxicity and safety profiles. Such investigations would provide a more comprehensive understanding of the potential therapeutic and nutritional applications of lycopene–PVP K30 formulations.

## 5. Conclusions

This study demonstrated that ball milling can be successfully used to produce amorphous lycopene–PVP K30 systems with superior physicochemical and biological properties compared to crystalline lycopene. XRPD, DSC, and FTIR analyses unequivocally confirmed the complete amorphization of lycopene in the polymer carrier, accompanied by potential intermolecular interactions that stabilize the dispersions.

Evaluation of functional properties revealed that incorporating lycopene into the PVP K30 matrix significantly increased its water solubility and antioxidant activity. Among the formulations tested, systems containing 5% and 10% lycopene exhibited the most favorable profiles. The 10% formulation demonstrated the highest initial solubility and radical scavenging capacity, while the 5% formulation demonstrated greater stability over time. In contrast, the 15% system was characterized by reduced solubility and antioxidant activity, likely due to aggregation or precipitation at higher lycopene concentrations.

In summary, these results indicate that a lycopene content of 5–10% in PVP K30 provides the most favorable balance between solubility, stability, and antioxidant activity. The results confirm that lycopene was molecularly dispersed within the amorphous PVP K30 matrix, forming a solid solution rather than a simple solid dispersion. These findings highlight the potential of ball-milled lycopene–PVP K30 systems as promising candidates for future applications in food supplements and pharmaceutical formulations aimed at improving lycopene bioavailability and functionality.

## Data Availability

The original contributions presented in this study are included in the article/Supplementary Materials. Further inquiries can be directed to the corresponding author.

## Supplementary Materials

The following supporting information can be downloaded at https://www.mdpi.com/article/10.3390/polym17212916/s1: Figure S1: Fragments of the lycopene structure marked in blue are associated with band vibrations at (a) 613 cm^−1^—CCC scissoring + CH_2_/CH_3_ rocking; (b) 806 cm^−1^—CH twisting; (c) 824 cm^−1^—CH rocking and wagging; (d) 880 cm^−1^—CC stretching + CH wagging; (e) 959 cm^−1^—CH wagging and rocking; (f) 1551 cm^−1^—CH scissoring, wagging, and twisting. Assignments are based on theoretical FT-IR DFT spectra.
