# Supplementary material for: Amorphous Lycopene–PVP K30 Dispersions Prepared by Ball Milling: Improved Solubility and Antioxidant Activity
Source: Polymers (Basel). 2025 Oct 31;17(21):2916. doi: 10.3390/polym17212916 (PMC12609523; doi:10.3390/polym17212916)
Supplement: Supplementary file 1 [file polymers-17-02916-s001.zip › polymers-3929901-supplementary.pdf]

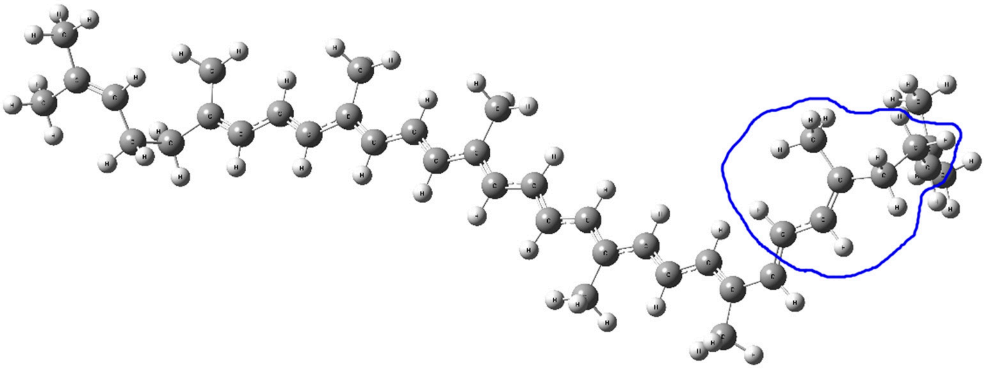

(a)

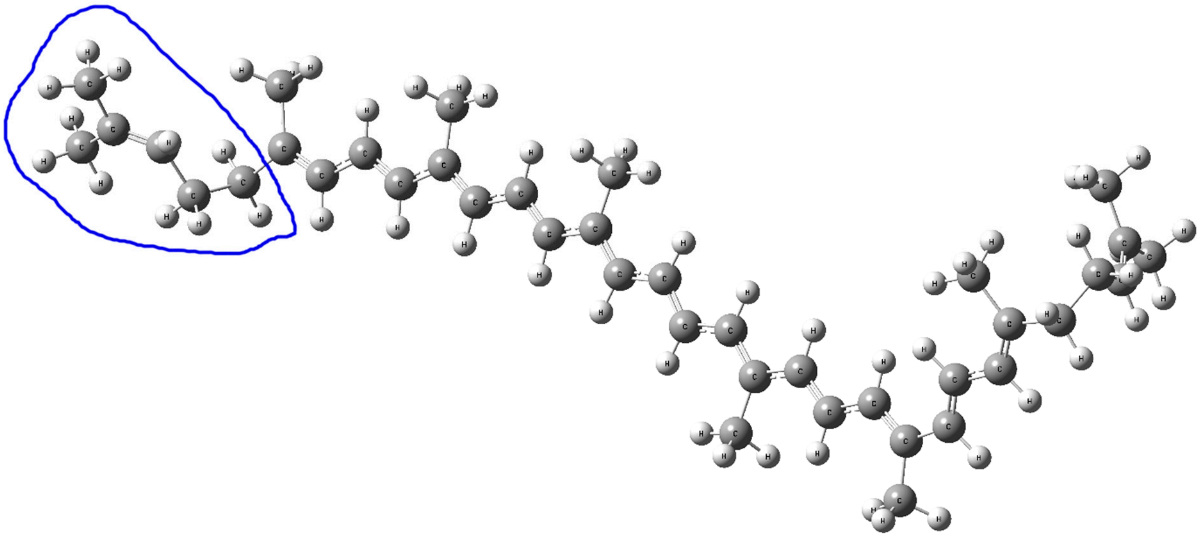

(b)

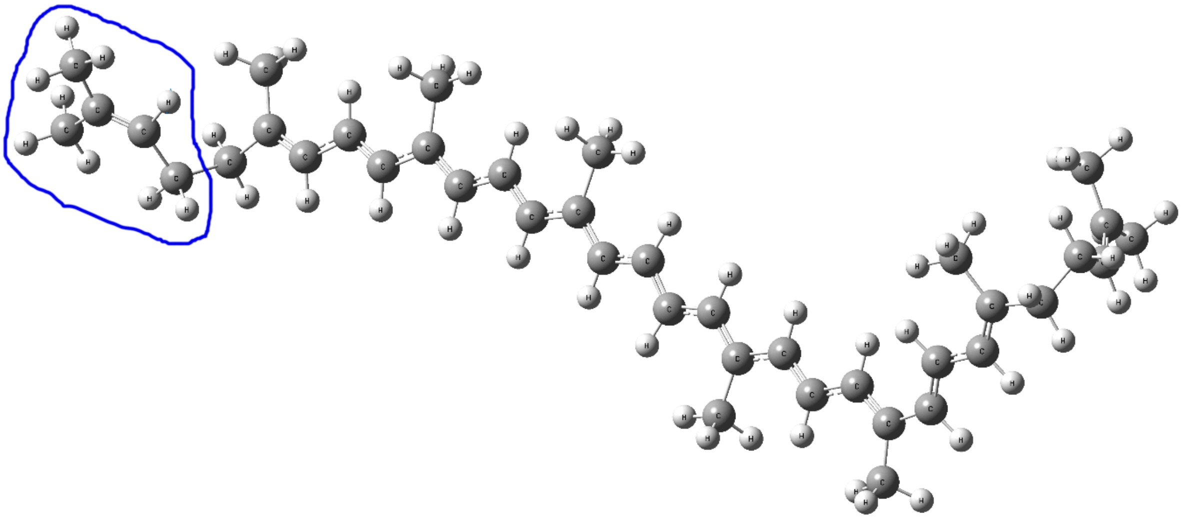

(c)

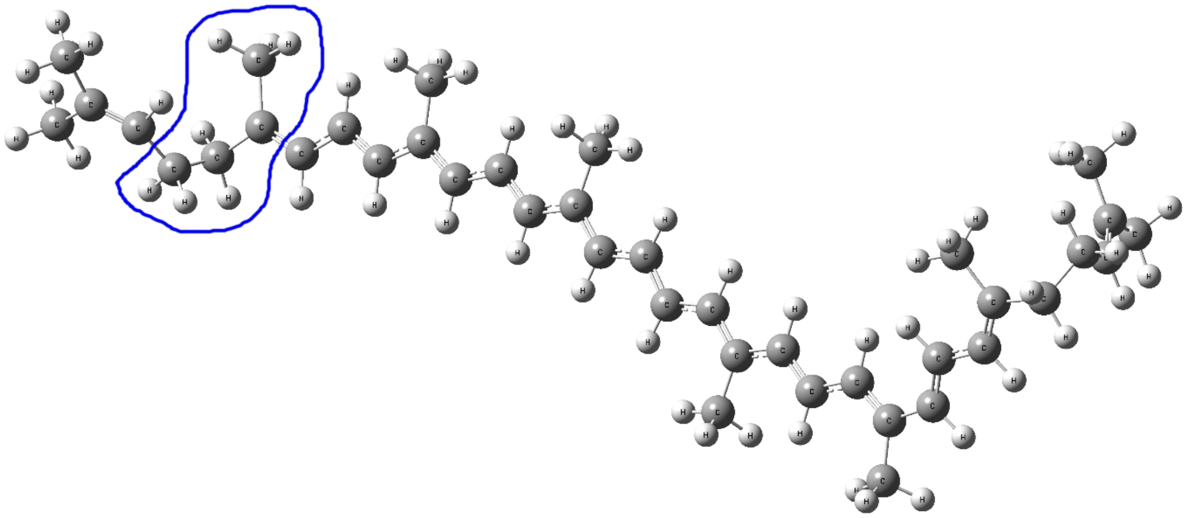

(d)

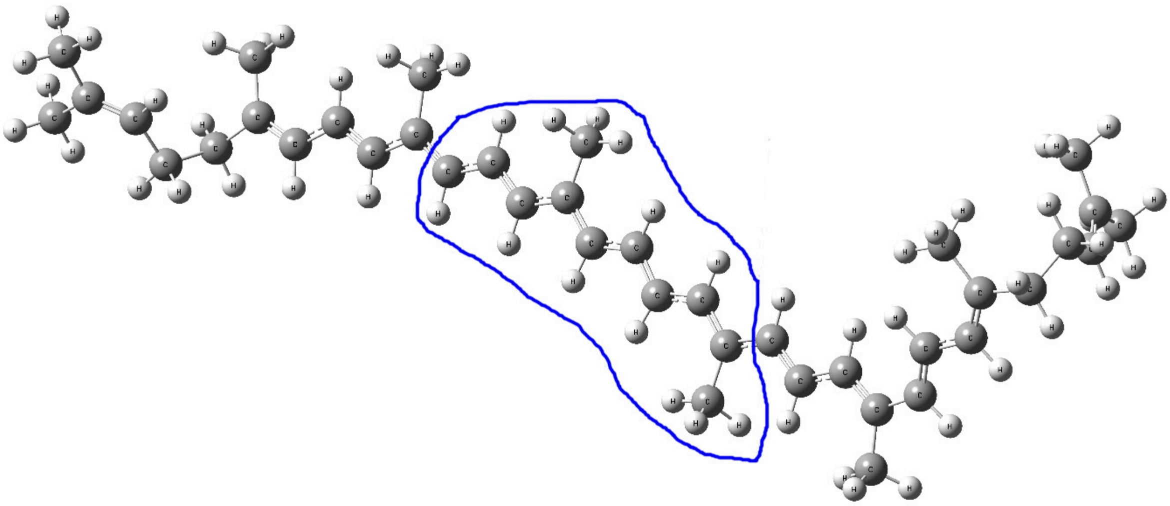

(e)

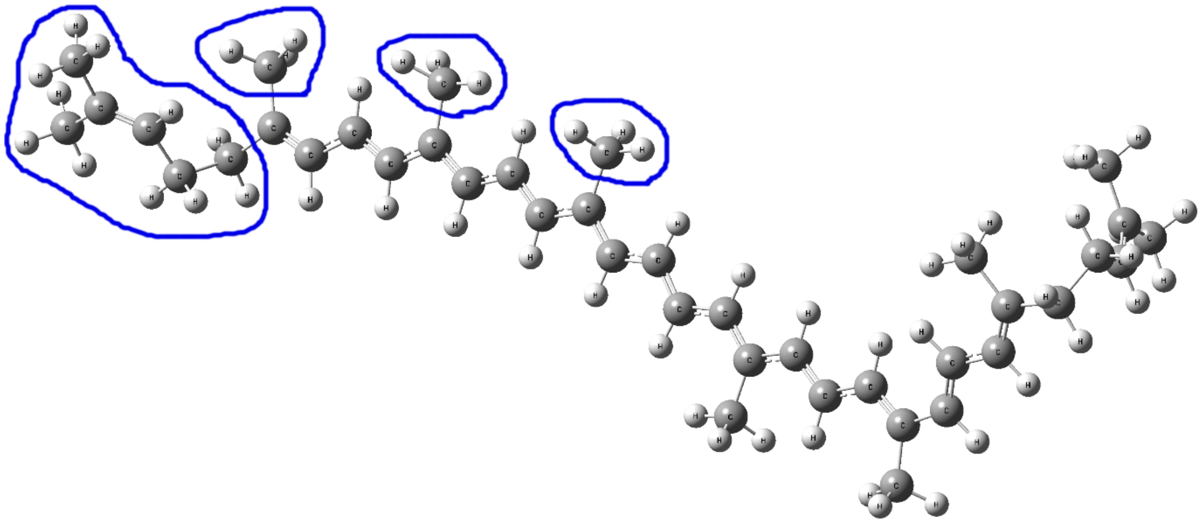

(f)

Figure S1. Fragments of the lycopene structure marked in blue are associated with band vibrations at (a) 613 cm^{-1} – CCC scissoring + CH_2/CH_3 rocking, (b) 806 cm^{-1} – CH twisting, (c) 824 cm^{-1} – CH rocking and wagging, (d) 880 cm^{-1} – CC stretching + CH wagging, (e) 959 cm^{-1} – CH wagging and rocking, (f) 1551 cm^{-1} – CH scissoring, wagging, and twisting. Assignments based on theoretical FT-IR DFT spectra.
